# The Intestinal Macrophage–Intestinal Stem Cell Axis in Inflammatory Bowel Diseases: From Pathogenesis to Therapy

**DOI:** 10.3390/ijms26072855

**Published:** 2025-03-21

**Authors:** Tao Quan, Ran Li, Ting Gao

**Affiliations:** College of Veterinary Medicine, China Agricultural University, Beijing 100083, China; s20243051072@cau.edu.cn (T.Q.); 18246269672@163.com (R.L.)

**Keywords:** intestinal stem cells, inflammatory bowel disease, macrophage, immunometabolism

## Abstract

The gut plays a crucial role in digestion and immunity, so its balance is essential to overall health. This balance relies on dynamic interactions between intestinal epithelial cells, immune cells, and crypt stem cells. Inflammatory bowel disease (IBD), which consists of ulcerative colitis and Crohn’s disease, is a chronic relapsing inflammatory disease of the gastrointestinal tract closely related to immune dysfunction. Stem cells, known for their ability to self-renew and differentiate, play an important role in repairing damaged intestinal epithelium and maintaining homeostasis in vivo. Macrophages are key gatekeepers of intestinal immune homeostasis and have a significant impact on IBD. Current research has focused on the link between epithelial cells and stem cells, but interactions with macrophages, which have been recognized as attractive targets for the development of new therapeutic approaches to disease, have been less explored. Recently, the developing field of immunometabolism has reinforced that metabolic reprogramming is a key determinant of macrophage function and subsequent disease progression. The aim of this review is to explore the role of the macrophage–stem cell axis in the maintenance of intestinal homeostasis and to summarize potential approaches to treating IBD by manipulating the cellular metabolism of macrophages, as well as the main opportunities and challenges faced. In summary, our overview provides a framework for understanding the critical role of macrophage immunometabolism in maintaining gut health and potential therapeutic targets.

## 1. Introduction

The intestinal tract is a large and complex organ that is responsible for the digestion and absorption of nutrients, elimination of wastes, and immunomodulation. Coordination between multiple cells, including intestinal epithelial cells, immune cells, and stem cells, plays a key role in regulating homeostasis in the gut. Inflammatory bowel disease (IBD), including ulcerative colitis (UC) and Crohn’s disease (CD), is a chronic and recurrent multifactorial disease that originates within the gastrointestinal tract. Currently, IBD has become a major healthcare burden globally and is also recognized as a major pathological risk factor for the induction of colorectal cancer. Therefore, better parsing the pathophysiologic basis and molecular or biochemical processes of IBD may provide new perspectives for our early diagnosis and discovery of appropriate therapeutic options [1].

Macrophages are first-line cells of innate immunity, located in the gut lamina propria and mesenchymal tissues, and are responsible for early inflammation onset and late inflammation relief. In the last decade or so, with the burgeoning field of immunometabolism, and the mechanisms of interactions between immunity and metabolism, researchers have emphasized that cellular metabolism has emerged as a key factor in the activation of macrophages and the regulation of their function [2,3]. Notably, dysregulation of macrophage immunometabolism is not only recognized as a key determinant of intestinal diseases such as IBD but also plays an important role in a variety of diseases including cancer, cardiovascular disease, atherosclerosis, and lipid metabolism imbalance. Therefore, targeting macrophage immune metabolism with small molecules and metabolic intermediates may provide new perspectives for the treatment of IBD [4,5,6]; however, the metabolic profile of macrophages in the context of IBD has not been fully revealed.

LGR5+ stem cells are located in the basal layer of the intestine and possess a strong capacity for self-renewal and differentiation. Intestinal stem cells maintain homeostasis in the gut by continuously generating intestinal epithelial cells and a variety of other cell types to replenish cell death caused by normal or abnormal factors in the gut. It has been shown that a complex network of signaling pathways, including BMP, Wnt/β-linker protein, Notch, and Hedgehog, facilitates communication between crypt stem cells and epithelial cells that work together to maintain intestinal tissue homeostasis [7]. However, these do not fully elucidate the regulatory mechanisms that maintain stem cell homeostasis.

In this review, we generalize and summarize the role of the macrophage–stem cell axis in regulating intestinal homeostasis and the interactions between these two to elucidate their roles in maintaining the intestinal mucosal barrier, modulating immune responses and promoting tissue repair. In addition, we provide a framework to understand the metabolic profile of macrophages in IBD. We also highlight the salient features of macrophage immune metabolic pathways in IBD and summarize the potentially effective therapeutic options proposed for targeting macrophage immune metabolism in IBD, as well as the opportunities and challenges for future development. Our goal is to provide new insights and strategies for the prevention and treatment of gut-related diseases.

## 2. Intestinal Homeostasis and Inflammatory Bowel Disease

The intestinal tract is anatomically divided into the small intestine, which includes the duodenum, jejunum, and ileum, and the large intestine, which includes the cecum, colon, and rectum, and these segments of the intestinal tract collectively perform the roles of nutrient digestion and absorption, waste elimination, and immune regulation. In addition, the intestinal tract is also the first barrier for the host to play a defense role. The small intestine relies on the intestinal epithelium to digest and absorb nutrients, while the intestinal epithelium can also be regarded as a mucosal barrier against the invasion of toxic and harmful substances from the outside world. Below the epithelium is a thin layer of connective tissue known as the lamina propria, which plays a major role in intestinal immunity. Various types of cells in the intestine, immune cells (including macrophages, dendritic cells, T cells and B cells, etc.) and intestinal epithelial cells (IEC) are independent of each other and closely linked, and together they maintain intestinal homeostasis [8,9]. In addition, intestinal health is also closely related to the overall health, nutritional status, intracirculatory homeostasis, and psychological status of the body [10,11]. Therefore, an imbalance in intestinal homeostasis cannot induce a variety of intestinal diseases, such as IBD (CD and UC), diarrhea, intestinal cancer, etc., and can easily lead to problems in other tissues and organs of the organism [12].

IBD, including CD and UC, is one of the most common intestinal diseases, and has been referred to as the “green cancer” due to its unknown etiology and difficulty in treating the disease [13]. IBD, which is typically characterized by diarrhea, abdominal pain, and intestinal bleeding, is a pathological process caused by damage to the intestinal mucosal barrier, an imbalance of intestinal microbiota, and an immune disorder [14,15]. IBD is a widespread disease with a global prevalence, especially in Western countries, and its incidence is increasing [16]. Although the predisposing factors and pathologic process of IBD are not clear, genetic factors, dietary habits, and environmental factors are believed to play important roles in the pathogenesis of IBD [17,18]. Among them, intestinal immune dysfunction is regarded as one of the important factors that induce IBD. Specifically, intestinal immune cells, including macrophages, dendritic cells, T cells, and B cells, can maintain intracellular homeostasis by secreting a variety of cytokines [19], and alterations in these cytokines can induce intestinal inflammation, which is closely related to the pathogenesis of IBD [20,21]. In addition, intestinal flora can provide key signaling molecules during the development and functioning of immune functions. Therefore, intestinal flora and their metabolites are not only involved in the regulation of intestinal immune processes but can also influence the susceptibility of the host to multiple immune-mediated diseases, including IBD [22,23], despite the gut microbiota–immunity–IBD axis and its complexity. The relationship between the triad of gut microbes, host immunity, and IBD has been discussed and summarized in some detail in several refereed journals.

New research suggests that improving the inflammatory response may be a new and effective target for treating patients with IBD [24,25,26]. Data consistent with this study show that a biologic agent, an inhibitor of tumor necrosis factor (TNF)-α, effectively accelerated the rate of mucosal healing in patients with IBD, signaling great progress in the treatment of IBD [19]. However, there are some patients who do not respond significantly to such agents, which means that new therapies need to be sought to repair mucosal damage in IBD patients. Earlier studies have suggested that transplantation or infusion of mesenchymal stem cells (MSCs) and hematopoietic stem cells (HSCs) into IBD patients can have beneficial effects, and a series of trials have been conducted to validate the effectiveness of this therapy [27,28]. Not only that, but recent clinical data also suggest that allogeneic bone marrow MSCs are still capable of treating patients with celiac disease in the cavities [29]. Thus, whether stem cells can be a good alternative treatment for patients with IBD remains controversial, but great potential exists.

## 3. How Immune Cells Modulate Intestinal Stem Cells to Ameliorate Intestinal Inflammation

It is well known that intestinal stem cells (ISC) have a strong proliferative and differentiation capacity, which allows them to continuously replenish dead and detached intestinal epithelial cells and to generate organoids by in vitro culture [30]. The resulting organoids are structurally very similar to intestinal epithelial cells, with intestinal villi and crypts and contain a variety of cell types from the intestinal epithelium [31]. Transplantation of this intestinal organoid to mucosa suffering from inflammation is expected to accelerate healing of the injured mucosa [32]. In addition, there are also data suggesting that organoids cultured from intestinal stem cells extracted from fetal intestine or adult small intestine can also be successfully transplanted to damaged sites in the colon, but they are slightly less able to adapt to the environment [33]. Recent studies have confirmed that human intestinal organoids accelerate healing of damaged mucosa in immunodeficient mice [34]. Therefore, we believe that restoration of intestinal stem cell activity may provide force an effective therapeutic approach to re-establish the mucosal barrier in IBD and, thus, improve the clinical symptoms of IBD.

Multiple mucosal surfaces of the organism, such as the respiratory and gastrointestinal tracts, are in constant contact with each other and with a variety of commensal microorganisms and pathogens from inside and outside the body. In the intestine, a dense monolayer of epithelial cells bound by tight junction proteins prevents these toxic and harmful substances from entering the host. As a result of the continuous attack by harmful substances, this epithelial cell needs to renew itself continuously, approximately every 5–7 days [35].

However, when the renewal process of intestinal epithelial cells is impeded, it can lead to the development of a variety of intestinal diseases, such as IBD and/or colon/rectal cancer.

The differentiation of intestinal stem cells into different daughter intestinal epithelial cells is in a dynamic equilibrium process. During this process, various signaling pathways coordinate intercellular communication and work together to maintain intestinal homeostasis. Dysregulation of these pathways also predisposes to intestinal diseases. Currently, a large number of studies have highlighted several key signaling pathways by which intestinal stem cells promote repair and proliferation of intestinal epithelial cells, including the BMP signaling pathway, Wnt/β-catenin signaling, Notch signaling pathway, Hedgehog signaling pathway, Hippo-YAP signaling pathway, and EGF signaling pathway, but these studies have not been able to elaborate the specific mechanisms of regulating intestinal stem cells [36].

Previous data have demonstrated the importance of immune function in maintaining homeostasis in the gut [37]. New studies have found that immune cells and intestinal epithelial cells communicate with each other despite their relative independence, and that normal communication between these two is a key mediator in maintaining intestinal function [11,38,39]. Not only that, but immune cells are also involved in the regeneration process of IEC [11,40,41]. Increasing evidence has found that immune cells play an important role in the self-renewal of ISC. The in vitro intestinal stem cell-immune cell co-culture system provides effective evidence to argue the validity of this view.

Therefore, this section summarizes the important roles of multiple immune cells, including T cells, B cells, and macrophages, in regulating the ecological niche of ISC and their pathways of action.

### 3.1. T Cells

T cells present in the intestinal lamina propria are not only involved in intestinal immune function but have also emerged as effective targets for the treatment of immune dysregulation-mediated intestinal injury. Recent data suggest that both CD4^+^ and CD8^+^ T cells have the potential to mediate ISC luminal injury in graft-versus-host disease models. Following bone marrow transplantation (BMT)-mediated gastrointestinal tract injury, diseased T cells identified the ISC ecotone as the site of intestinal origin in three-dimensional imaging examinations [42]. In addition, recruitment of pathogenic T cells to the basal region of the crypt resulted in the loss of ISC expressing both major histocompatibility complex class (MHC) I and II [42]. In a similar report, another study explored changes in the ISC ecological niche using a T-cell-mediated in vivo model of graft-versus-host disease (GVHD) and in vitro intestinal organoid-T-cell co-cultures [43]. This study demonstrated that both ISC and Paneth cell numbers per crypt were significantly reduced in mice treated with bone marrow or allogeneic T cells. In contrast, these mice had significantly increased densities of both lamina propria- and intraepithelial-associated T cells in the crypt region [43].

Recently, researchers developed methods for co-culturing gut-like organs with T cells in human and mouse models to reveal the molecular mechanisms underlying the loss of ISC activity in IBD. It was found that mouse heterologous reactive T cells significantly reduced the number of intestinal-like organs, whereas human-derived CD8^+^ cytotoxic T cells significantly reduced the efficiency of intestinal-like organs. Meanwhile, in the absence of Pan cells, IFN-γ secreted by CD4^+^ T cells induced apoptosis in ISC by activating the JAK/STAT signaling pathway [41]. Overall, these studies have not only expanded our understanding of immune cell–intestinal stem cell communication, but based on this, have also suggested that T cells could be an effective target for ameliorating IBD by acting on ISC. In future studies, considering that T cells may act on ISC by secreting IFN-γ, inhibition of the JAK/STAT signaling pathway could be considered as a new ameliorative pathway for effective treatment of IBD.

Meanwhile, recent studies have utilized fetal intestinal organoids to explore the interactions between epithelial and immune cells during fetal development [44]. Recent single-cell RNA sequencing data identified a specific group of CD4^+^ effector memory T cells that secrete large amounts of TNF-α [45]. On this basis, the investigators used a co-culture model of organoids from fetal intestinal cultures with CD4^+^ Tem cells to explore the effect of TNF-α on intestinal epithelial cells. The results showed that a small number of CD4^+^Tem cells from fetal intestines mediated TNF-α to promote the proliferation of intestinal stem cells; conversely, a large number of CD4^+^Tem cells from fetal intestines disrupted the function of intestinal stem cells [45]. The latter result is consistent with observations in infants with necrotizing small bowel colitis [45].

Interaction between T helper (TH) cells and intestinal stem cells can help restore intestinal stem cell activity and rebuild the intestinal epithelial barrier system [40]. The current study utilized single-cell RNA sequencing to identify a subpopulation of Lgr5+ ISC that can highly express MHC II molecules. In vitro culturing of intestinal organoids revealed that MHC II-expressing intestinal stem cells have the ability to present antigens in the functioning of intestinal epithelial cells. Moreover, MHC II mediated the contact between TH cells and intestinal stem cells, which in turn induced the activation of TH cells in an antigen-presenting manner [38]. Additional studies have also observed the effects on intestinal stem cells after co-culture of intestinal organoids with different TH cell subsets or after specific labeling with cytokines to better reveal the interactions between intestinal stem cells and TH cells. It was found that the addition of TH2 cells or IL-14 with IL-13 cytokines depleted the intestinal stem cell pool. Consistent with this, TH1 and TH17 cells or their secreted cytokines IFN-γ and IL-17 significantly reduced the number of intestinal stem cells but upregulated the number of transit-expanding cells. In contrast, regulatory T (Treg) cells or IL-10 upregulated the number of intestinal stem cells [40]. Another study found that co-culturing human T cells with ISC promoted the maturation of human pluripotent stem cell-derived intestinal organoids (hIOs) in vitro, suggesting that IL-2 promotes the growth of human T cells by activating the STAT3 signaling pathway [46].

Together, the above findings suggest that interactions between intestinal stem cells and T cells are involved in regulating intestinal stem cell fate. Notably, intestinal stem cells can sense T cells via MHC II molecules and, thus, activate T cells. However, more studies are needed in the future to further reveal the relationship between intestinal immunity and the intestinal epithelial barrier.

### 3.2. Innate Lymphoid Cells

Innate lymphoid cells (ILCs) are regarded as important regulators of intestinal mucosal immunity and play an important role in the maintenance of intestinal homeostasis and the integrity of the intestinal mucosal barrier [47]. ILCs can be categorized into three major groups, namely, ILC1, ILC2, and ILC3, based on the cytokines they produce, their phenotype, and the developmental pathway [48]. It has been suggested that the cytokines secreted by ILCs are closely related to intestinal stem cell activity and function. Among them, IL-22 has been shown to be involved in regulating the maintenance of intestinal stem cell activity and differentiation and to protect against DNA damage [49,50]. Several studies have demonstrated that IL-22 promotes intestinal epithelial repair in DSS-induced colitis [51,52,53]. However, the exact mechanism of how IL-22 is involved in regulating the fate of intestinal stem cells remains to be further explored, although we already know that IL-22 receptors exist in intestinal stem cells [54,55]. In addition, IL-22 is closely associated with the repair of intestinal epithelial damage in GVHD and BMT, in which allogeneic T cells can inhibit ISC function [55,56]. Mechanistically, IL-22 can activate the STAT3 signaling pathway in Lgr5+ intestinal stem cells independently of PAN cells to maintain intestinal stem cell activity and promote their differentiation [56]. IL-22 not only enhances the survival of intestinal stem cells but also their proliferative viability, the latter of which is critical for the regeneration of damaged epithelial cells [55]. Together, the above studies suggest that the IL-22–STAT3 axis may be one of the effective targets for the treatment of IBD. However, a recent study seems to contradict this conclusion [57]. This study showed that in the colonic epithelium, IL-22 induces endoplasmic reticulum stress response with transcriptional programs. Specifically, high expression of the IL-22-responsive transcriptional module and endoplasmic reticulum (ER) stress response was found in CD patients. In contrast, blocking IL-22 alleviated colitis by inhibiting epithelial ER stress [57]. The above studies suggest that IL-22 acts as a double-edged sword in the pathogenesis of chronic intestinal inflammation.

Environmental genotoxic silver can also lead to mutations in intestinal stem cells and will furthermore induce cancer [58,59]. As an evolutionarily conserved response pathway in ISC, DNA damage response (DDR) can be involved in maintaining genome integrity at the cellular level [60]. Recent studies have suggested that IL-22, produced by ILC3 and gδ T cells in the intestinal mucosa, could be regarded as a potent regulator of the DDR pathway in colonic stem cells (CSCs) [61]. Specifically, after DNA damage, IL-22 can overexpress the IL-22 receptor, which in turn effectively activates the DDR in CSCs. Alternatively, when IL-22 signaling is inhibited, intestinal stem cells exposed to carcinogens show significant mutations, which can exacerbate colon cancer development and progression [61]. In addition, a previous study explored the protective effect of Lactobacillus royale on intestinal mucosal integrity by establishing a co-culture system of intestinal organoids and lamina propria lymphocytes, and the results demonstrated that the Lactobacillus royale metabolite indole-3-aldehyde furthered enteric stem cell-mediated regeneration of intestinal epithelium by stimulating the secretion of IL-22 by lamina propria lymphocytes [50].

Group 2 innate lymphoid-like cells (ILC2) are also involved in the regulation of intestinal stem cell fate. Among them, IL-13 secreted by ILC2 has been shown to promote the differentiation of cup cells in intestinal organoid models. Also, IL-33 secreted by intestinal epithelial cells promotes IL-13 secretion by ILC2 [62]. In addition, it has been shown that in the intestine, IL-4 and IL-13 can be involved in the differentiation of tufted cells that regulate type II immune responses [63,64,65]. It is interesting to know about the relationship between the tufted cell-mediated intestinal immune system and epithelial cells. For example, tufted cells may act as immune sentinels and respond accordingly to parasites present in the gut. In addition, succinic acid from helminths induces IL-25 secretion by tufted cells [65,66], whereas IL-22 recruits ILC2 in the intestinal mucosa and secretes large amounts of IL-4 with IL-13, inflammatory factors that, in turn, clear parasites by promoting mucus production by cuprocytes [65]. Although these studies suggest that IL-4 and IL-13 secreted by ILC2 can induce more cell differentiation, including cup and tuft cells, the interactions between ILC2 and intestinal stem cells and their specific molecular mechanisms still need to be further explored.

Currently, a range of anti-inflammatory drugs (corticosteroids and aminosalicylates), immunosuppressive agents (azathioprine, methotrexate, and mercaptopurine), and biologic drugs (anti-TNFα drugs such as uterumab and vedolizumab; and the interleukin-12/23 antagonist uterumab) have been developed for the treatment of intestinal inflammation, targeting these above immune cells [17,67]. However, there are some limitations of these drugs that restrict their general use, such as limited efficacy, severe side effects, and unresponsiveness to immunosuppressive agents [17,67]. Over the past decades, targeting macrophages has been recognized as one of the measures that can effectively treat intestinal inflammation [4].

## 4. The Role of Macrophages in IBD

IBD is a major case of intestinal homeostatic imbalance and further pathological changes. Activation of the immune system with increased inflammatory response is an important hallmark of IBD, and macrophages are thought to play an important role in this process [68].

Furthermore, intestinal macrophages are involved in the regulation of intestinal stem cell renewal, homeostasis maintenance and differentiation. Studies have shown that macrophage depletion significantly reduces the number of intestinal stem cells and impairs the differentiation process of intestinal stem cells. Unlike macrophages in other tissues, intestinal macrophages are not only abundant but also play the first line of defense in the intestinal tract, and, therefore, intestinal macrophages have distinct anti-inflammatory characteristics and exhibit high phagocytic and bactericidal activity and high tolerance to foreign substances [69,70].

### 4.1. Origin, Phenotype and Function of Intestinal Macrophages

**Figure 1:**
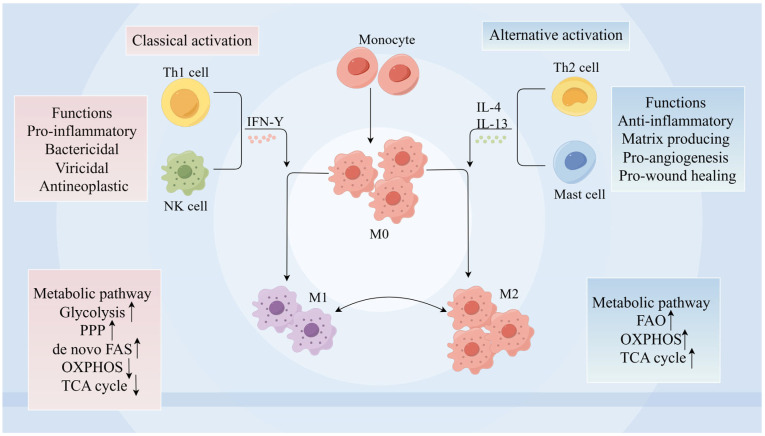
**Characteristics of polarized macrophages** M0 macrophages derived from monocyte polarizes into M1 macrophages with IFN-γ secreted from Th1 cell and NK cell stimuli. M1 macrophages perform pro-inflammatory, bactericidal, viricidal, and antineoplastic activity. In M1 macrophages, glycolysis, PPP, and de novo FAS are upregulated, and OXPHOS and the TCA cycle are downregulated. M0 macrophages polarizes= into M2 macrophages with IL-4, IL-10, and IL-13 secreted from Th2 cell and mast cell. M2 macrophages preform anti-inflammatory, matrix producing, pro-angiogenesis and pro-wound healing function. In M2 macrophages, FAO, OXPHOS, and the TCA cycle are upregulated. In special cases, M1 can be polarized toward M2. But whether M2 can be polarized to M1 is still debated. M0 macrophages: unactuated macrophages; M1 macrophage: classically activated macrophages; M2 macrophages: alternatively activated macrophages; IFN: interferon; IL: interleukin; PPP: pentose phosphate pathway; OXPHOS: oxidative phosphorylation; FAS: fatty acid synthesis; FAO: fatty acid oxidation; TCA: tricarboxylic acid cycle; the dots under the arrows are inflammatory factors; A rising arrow indicates a strengthening of the pathway and a falling arrow indicates a weakening of the pathway.

Macrophages are derived from circulating monocytes in the blood or from a pool of resident macrophages in the gut and are one of the important immune cells against foreign pathogens. Results of in vitro studies targeting monocyte-derived macrophages have shown that macrophages can be categorized into classically activated M1 macrophages and selectively activated M2 macrophages [71,72,73]. Although the use of M1 versus M2 macrophages can effectively differentiate macrophage response types, it does not fully summarize all phenotypes of macrophages in vivo [74,75,76]. Nevertheless, this categorization still contributes to our understanding of the metabolic programming of different macrophage functions. Therefore, in the description of this review, we refer to those that stimulate inflammation/tissue injury/hinder wound healing and trigger Th1/Th2 immune responses as M1 macrophages, and those that antagonize inflammation/promote tissue repair and promote Th2 immune responses as M2 macrophages (Figure 1).

### 4.2. Differentiation of Macrophages During IBD

In the maintenance of organismal homeostasis, monocytes derived from mouse circulating Ly6Chi and human CD14hi are continuously recruited into the intestine and further differentiate into mature CX3CR1hiF4/80+ macrophages to complement resident macrophages. These blood-borne macrophages exhibit an M2-like macrophage phenotype due to the high expression of CD206 and CD163, which can exert anti-inflammatory effects by secreting the anti-inflammatory factors IL-10 and TGF-β and decreasing the response to TLR [77]. In addition, this cell class is involved in the maintenance of intestinal homeostasis by effectively clearing apoptotic epithelial cells and translocated bacteria [78]. In addition, M2 macrophages maintain the integrity of the intestinal epithelial barrier by promoting IL-10 secretion from CD4^+^CD25^+^ Treg [79], which in turn promotes intestinal stem cell renewal [80,81].

**Figure 2:**
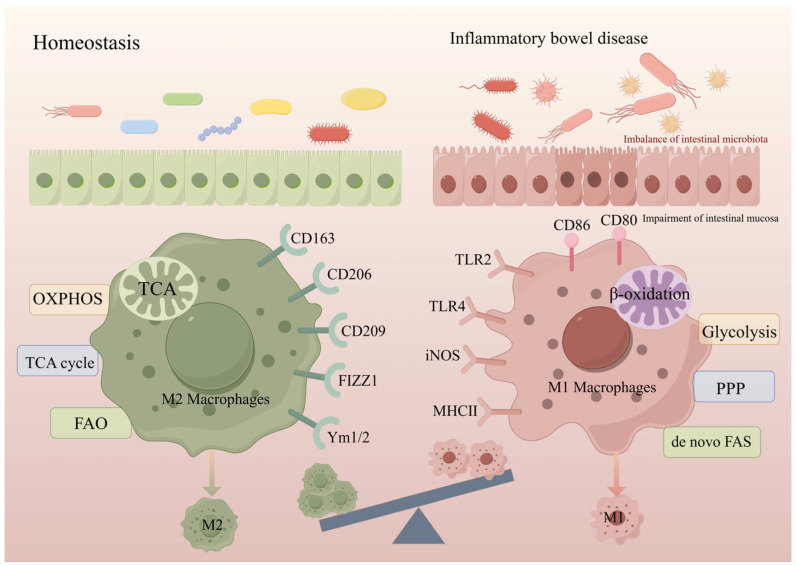
**Macrophage status in the healthy gut versus under IBD.** In the context of intestinal homeostasis, the intestinal mucosal barrier is intact, and the intestinal flora is balanced. In the lamina propria, M0 macrophages are predominantly polarized to M2 macrophages. Its surface markers include CD163, CD206, CD209, FIZZ1, and Ym1/2, and the metabolic mode of M2 macrophages is dominated by OXPHOS, the TCA cycle, and FAO. On the background of inflammatory bowel disease, the intestinal mucosal barrier is damaged and the intestinal flora is imbalanced. In the lamina propria, it is dominated by M1 macrophages, whose surface markers include CD86, CD80, TLR2, TLR4, iNOS, and MHC II, and whose metabolism is dominated by glycolysis, PPP and de novo FAS; CD163, CD206, CD209, FIZZ1, Ym1/2: Marker proteins of M2 macrophages; CD80, CD86, TLR2, TLR4, iNOS, MHC II: Marker proteins of M1 macrophages.

Cellular metabolism can be involved in macrophage activation and function by generating energy and building blocks required for cell maintenance and proliferation [82,83]. The major metabolic pathways involved in macrophage immune metabolism include glycolysis, tricarboxylic acid cycle (TCA), pentose-phosphate pathway (PPP), fatty acid oxidation (FAO), fatty acid synthesis, and amino acid metabolism [82,83,84]. Notably, macrophage subpopulations present different metabolic and energy requirements, as well as the flexibility to respond to different environments and cytokines [85] (Figure 2).

Specifically, the metabolism of M1 macrophages activated by lipopolysaccharide is characterized by an impaired TCA and mitochondrial oxidative phosphorylation (OXPHOS), in contrast to enhanced glycolytic pathways and increased PPP flux [82,86,87,88]. These metabolic pathways rapidly produce ATP and accumulate large amounts of immunomodulatory molecules, including lactate, citrate, and succinate [82]. In contrast, IL-4-activated M2 macrophages maintain an intact TCA cycle and full OXPHOS [89,90,91]. Whereas amino acid metabolic pathways are also involved in macrophage polarization, e.g., glutamine is required for IL-1β secretion by M1 macrophages [88,92], M2 macrophage polarization requires the glutamine-UDP-N-acetylglucosamine pathway and α-ketoglutarate produced by glutamine catabolism for maintenance [92,93]. In addition, arginine is required for the substrate of iNOS and arginase in M1 versus M2 macrophages, respectively [90,94]. Importantly, macrophage polarization in vivo is also stimulated by a variety of nutrients, but since the supply of nutrients is very limited, macrophages also need to compete with other cells for nutrients [95]. Therefore, when nutrient metabolism is altered, macrophages must promptly adjust their immune response to meet the needs of the organism. In the next section, we will generalize and summarize how perturbations in intracellular nutrient metabolism affect macrophage polarization and function during the pathological process of IBD.

## 5. How Macrophages Regulate Intestinal Stem Cells to Maintain Intestinal Homeostasis

The successful establishment of an intestinal organoid and macrophage co-culture system confirmed the involvement of macrophages in the functional regulation of ISC. Macrophages rely on a variety of cytokines secreted by themselves to regulate intestinal stem cell function, including IL-6, IL-8, IFN-γ, and TGFβ-1, which are mediated by macrophages to promote the maturation of intestinal epithelial cells and the establishment of the mucosal barrier [96]. In the healthy intestine, macrophages are distributed at the crypts. Hyaluronic acid in the extracellular matrix can promote proliferation of intestinal stem cells by binding to TLR4 receptors on macrophage membranes first, which in turn promotes macrophage secretion of prostaglandin E2 (PGE2), which subsequently promotes proliferation of intestinal stem cells by activating EGFR signaling in intestinal stem cells, ultimately lengthening the length of the early intestine. When macrophages were depleted with chlorophosphate, the proliferative vigor of intestinal stem cells was significantly reduced, but no increase in the number of SOX9+ reserve stem cells was observed [97]. In contrast, the results of another study showed a significant decrease in the number and proliferative capacity of Lgr5+ intestinal stem cells but a significant increase in the proliferative viability of SOX9++ Bmil+ reserve stem cells after depletion of macrophages by long-term administration of CSF1R antibody in mice. This may be due to the difference in age between neonatal and adult mice.

Macrophages are also involved in the functional regulation of intestinal stem cells in the MSCs population. MSCs can ameliorate IBD by replacing damaged cells by homing to damaged tissues and differentiating into functional cells [98]. A study in mice showed that oral administration of *Lactobacillus rhamnosus* was protective of the gut and that *Lactobacillus rhamnosus* GG (LGG) was dependent on TLR2 and COX2 for its protective effect. It was associated with a shift in COX-2+ MSCs from the lamina propria of the villi to the lamina propria near crypt epithelial stem cells. This shift attenuated radiation-induced intestinal damage and accelerated the proliferation of intestinal stem cells [99].

Using a mouse model of IBD, macrophages were found to accelerate intestinal mucosal barrier repair by activating the Wnt signaling pathway [100]. Recent data further suggest that colony-stimulating factor (CSF1)-dependent macrophages in the intestinal wall can be used to maintain the ecological niche of intestinal stem cells in the small intestine [97], a result that suggests that CSF1 is expected to be a potent target for improving the intestinal epithelial barrier after inflammatory or chemotherapeutic injury. In addition, the researchers established a human intestinal-like-macrophage co-culture model to explore the interactions between the organism and intestinal pathogens [96]. The results of this study suggest that macrophages may play a role in promoting intestinal epithelial maturation and rebuilding the intestinal mucosal barrier by mediating their secretion of various cytokines such as IL-6, IL-8, IFNg, and TGFβ1 [96]. These studies have greatly expanded our understanding of the immune cell–intestinal stem cell interactions, but the reality is far more complex than we can comprehend.

In this review, we draw a framework for a better understanding of the metabolic signature of macrophages in IBD and summarize and generalize how this metabolic reprogramming process is triggered by microenvironmental factors, including signals originating from the host cell as well as sensing by microorganisms and their metabolites. We also outline the salient features of macrophage immune metabolism in IBD and summarize the therapeutic tools developed to target macrophage immune metabolism in IBD. Finally, we have also attempted to present the current problems and future developable directions in the field of studying macrophage immune metabolism and IBD.

**Figure 3:**
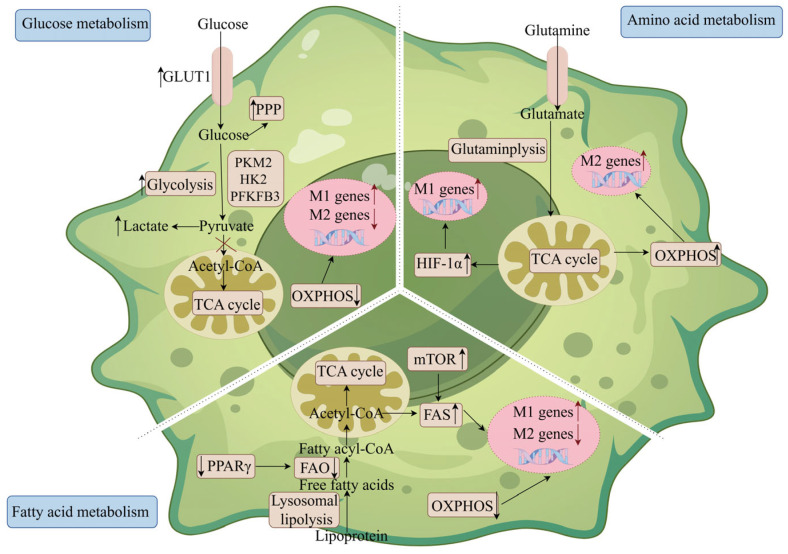
**Metabolic reprogramming of macrophages in IBD.** During IBD, three types of metabolism involved in macrophages, including glucose metabolism, amino acid metabolism, and fatty acid metabolism, are altered. In glucose metabolism, the entry of pyruvate into mitochondria is reduced and pyruvate is mainly converted to lactate, leading to a truncated TCA cycle, further promoting M1 macrophage-associated gene expression and inhibits M2 macrophage-associated gene expression. In fatty acid metabolism, the anti-inflammatory PPAR-γ and FAO are downregulated during IBD, which causes impaired OXPHOS and M2 macrophage polarization; supplementation of unsaturated fatty acids such as conjugated linoleic acid prevents IBD by targeting at PPAR-γ. In amino acid metabolism, glutamine is another key metabolic fuel for macrophages, and glutamine can enhance M2 polarization by fueling TCA cycle activity and OXPHOS or M1 polarization by activating succinate-HIF-1α pathway. FAO, fatty acid oxidization; FAS, fatty acid synthesis; GLUT1, glucose transporter 1; HIF-1α, hypoxia-inducible factor-1 alpha; HK2, hexokinase II; mTOR, mammalian target of rapamycin complex; OXPHOS, oxidative phosphorylation; PFKFB3, 6-phosphofructo-2-kinase/fructose-2, 6-bisphosphatase; PKM2, pyruvate kinase 2; PPAR-γ, peroxisome proliferator–activated receptor gamma; TCA, tricarboxylic acid; the dots under the arrows are inflammatory factors; A rising arrow indicates a strengthening of the pathway and a falling arrow indicates a weakening of the pathway.

In IBD, the process of M2-like macrophage polarization is inhibited, whereas the process of M1-like macrophage polarization is promoted [70,101]. A variety of inflammatory factors secreted by M1 macrophages, such as IL-12, IL-23, and IL-1β, mediate the inflammatory response triggered by Th1 and Th17, which further contribute to intestinal epithelial injury [102,103]. In particular, although M1 macrophages play a key role in the pathological colonization of colitis, they can still exert anti-inflammatory effects by promoting M2 macrophage polarization thereby accelerating the healing of mucosal injury [77,80]. Notably, it has been found that bone marrow-derived M2 macrophages significantly ameliorate colitis in mice by transplantation or intraperitoneal injection [104,105,106]. Since the balance between M2-M2 macrophages plays a critical role in intestinal health, unraveling the regulatory mechanisms of macrophage polarization will provide effective protocols for developing targeted macrophages to ameliorate intestinal inflammation (Figure 3).

### 5.1. Macrophage-Derived Factors Could Regulate Stem Cell Fate

Macrophages play a critical role in mammalian development and exhibit important homeostatic activity in virtually all organ systems of the body through the production of growth factors and other mediators that provide nutritional support to the tissues in which they reside [107]. Resident tissue macrophages are important drivers of inflammatory and tissue regenerative responses to infection, autoimmune disease, mechanical or toxic injury, and a variety of other causes of organ damage [108]. Following tissue injury, the basement membrane is further disrupted by macrophage-derived matrix metalloproteinases, which help to promote the movement of inflammatory cells toward the site of tissue injury. They also produce chemokines that direct the initial recruitment of inflammatory cells [109]. Macrophages can also act as scavenger cells, phagocytizing cellular debris, invasive biocytes, neutrophils, and other apoptotic cells present after tissue injury [110,111]. Thus, if macrophage recruitment or activation is disrupted after tissue injury, the early inflammatory response tends to be reduced [112]. However, impaired macrophage recruitment and activation may also reduce wound debridement and prolong exposure to pro-inflammatory stimuli, leading to incomplete tissue regeneration [113]. Thus, the repair response of macrophages after tissue injury must be well coordinated.

In addition to supporting the initial inflammatory response following injury, local tissue macrophages respond to stimuli found in the local environment and rapidly transition to a wound repair phenotype. Wound repair macrophages are characterized by enhanced production of multiple growth factors, including platelet-derived (PDGF), insulin-like (IGF-1), and vascular endothelial (VEGF-α) growth factors, which promote cellular proliferation and vascular development, collectively contributing to the relief of local hypoxia that occurs after injury [114,115,116,117,118]. They also produce soluble mediators, such as TGF-β1, which stimulate local and recruited tissue fibroblasts to differentiate into myofibroblasts, promoting wound contraction and closure, as well as synthesis of extracellular matrix components [119]. Proliferation and expansion of many neighboring parenchymal and stromal cells are also enhanced by macrophages, which may activate stem cells and local progenitor cell populations involved in the tissue repair response if the injury is severe or chronic [120].

During the final stages of tissue repair, macrophages exhibit an anti-inflammatory or pro-catabolic phenotype [121]. These macrophages preferentially secrete anti-inflammatory mediators such as IL-10 and TGF-β1 and express cell surface receptors such as PD-L2 to suppress the immune system and ameliorate inflammation, which, if not effectively controlled, leads to collateral cell death and prolongs or significantly impairs the repair process [122,123,124,125]. In some tissues, they may also replace or supplement the resident tissue macrophage population. Thus, the three major phases of tissue repair must be carefully regulated, with macrophages playing unique and critical roles at each stage [108]. Indeed, interruption of macrophage recruitment or activation at any point in the process may result in inefficient or incomplete repair, with uncontrolled production of macrophages by inflammatory mediators and growth factors and defective wound healing or macrophages both leading to an inefficient repair response. In some cases, dysregulation of the wound repair response may lead to the development of pathologic fibrosis or scar formation, which can further compromise normal tissue function and ultimately lead to organ failure and death [126]. Although tissue repair is orchestrated by multiple cell types since macrophages are involved in all stages of the wound repair response [71], they have emerged as potentially important therapeutic targets [127,128].

### 5.2. Macrophage Glucose Metabolism and IBD

During the pathologic process of IBD, one of the key pathological changes in IBD occurs due to an imbalance between oxygen supply and demand in the intestinal microenvironment, which further leads to severe hypoxia at the site of inflammation [129]. In this hypoxic environment, in order to meet the high energy demands of the gut, macrophages undergo a shift in the metabolic pathway from oxidative phosphorylation to aerobic glycolysis. This transition is mediated by hypoxia-inducible factor 1α (HIF-1α) and key glycolytic proteins including glucose transporter 1 (GLUT1), hexokinase II, PKM2, and 6-phosphofructo-2-kinase/fructose-2,6-bisphosphatase (PFKFB3) [89,130]. The HIF-1α-mediated glycolytic metabolic pathway plays a key role in the pro-inflammatory effects exerted by macrophages [131]. In addition, activation of HIF-1α in myeloid cells exacerbates the manifestation of colitis [132], whereas specific knockdown of the gene in myeloid cells significantly ameliorates colitis [133]. In addition, the glycolytic reprogramming process can be regulated through the formation of PKM2-HIF-1α complexes in the nucleus [134]. Recent findings showed that PKM2 de-adhesion of SIRT5 significantly reduced the levels of IL-1β produced by macrophages, which in turn ameliorated dextran sulfate sodium salt (DSS)-induced colitis in mice [130], suggesting that PKM2 could be an effective target for the treatment of IBD. Collectively, these findings explain that metabolic reorganization to aerobic glycolysis is a key feature that determines macrophage function and subsequent IBD progression.

In addition to enhanced glycolysis, M1 macrophages exhibit interrupted tricarboxylic acid cycle pathways, which can further lead to a high accumulation of succinate and citrate [86,88]. Succinate is not only an intermediate product of the TCA but also a receptor ligand for succinate receptor 1 (SUCNR1) [135]. It is important to note that IBD patients have significantly higher fecal and serum concentrations of succinate [136,137], and their intestinal expression of SUCNR1 is also significantly increased [138]. Importantly, large amounts of succinic acid promote the stabilization of intracellular HIF-1α, which in turn enhances glycolytic metabolism in M1 macrophages and ultimately induces the secretion of the pro-inflammatory factor IL-1β [88,139]. Studies on colitis have demonstrated that resting peritoneal macrophages from SUCNR1-deficient mice secrete less pro-inflammatory factors IL-1β, IL-6 and TNF-α and, thus, exert a protective effect against colitis. The second breakpoint in the TCA cycle occurs at isocitrate dehydrogenase, which leads to the accumulation of intracellular citrate, which subsequently moves from the mitochondria into the cytoplasm, where it is then cleaved by ATP-citrate lyase [140] This export and catabolism of mitochondrial citrate is essential for the secretion of prostaglandins, NO, and ROS in pro-inflammatory macrophages [140,141]. In addition, metabolomics analyses have mentioned that the levels of lactate, pyruvate, and citrate are all significantly increased in the serum of mice with spontaneous colitis [142], suggesting that the glycolytic process is enhanced with the blockage of the TCA cycling process, which is tightly linked to the pathogenesis of IBD. Therefore, succinate and citrate are considered as key metabolites that differentiate between IBD patients and healthy individuals.

### 5.3. Macrophage Fatty Acid Metabolism and IBD

Studies have also identified changes in lipid metabolism that are also present in IBD patients, which in turn could lead to the use of inflammatory lipids as a potential biomarker for IBD [143,144], where peroxisome proliferator-activated receptor gamma (PPAR-γ) could serve as a key conjugate of lipids with innate immunity [145,146]. Studies have shown that the activation of PPAR-γ in macrophages significantly ameliorates experimental colitis in mice [147], whereas specific knockdown of the PPAR-γ gene in macrophages exacerbates colitis pathology [148]. This result could partially explain the PPAR-γ-dependent lipid metabolism in macrophages due to the fact that activation of PPAR-γ could ultimately contribute to the metabolic reorganization of the anti-inflammatory phenotype of macrophages through upregulation of Arg1 expression, promotion of β-oxidized fatty acids, and mitochondrial biogenesis processes [149]. However, inactivation of the mTOR-Semaphorin 6D-PPAR-γ signaling pathway can lead to impaired fatty acid uptake and FAO processes, which in turn induces an imbalance in macrophage polarization and worsens the pathological changes in colitis [149]. Consistent with these findings, the utilization of dietary or probiotic-derived conjugated linoleic acid can further ameliorate IBD in both humans and mice through the activation of PPAR-γ [150]. Thus, together, these findings suggest that lipid metabolism is involved in macrophage polarization process and PPAR-γ can be considered as one of the effective targets for the treatment of IBD.

### 5.4. Macrophage Amino Acid Metabolism and IBD

In addition to protein building blocks, some amino acids are also involved in immunoregulation during macrophage metabolism. Not only that, disorders of amino acid metabolism have been observed in some IBD patients, and the degree of disorders is positively correlated with the severity of the disease [151,152]. Glutamine is not only the most abundant free amino acid in human plasma but also an essential amino acid required in the body during injury or disease processes [92], whereas patients with IBD often show altered metabolism of glutamine [153,154], including decreased glutamine concentration, decreased uptake, and decreased activity of glutaminase [154,155]. Glutamine metabolism plays a dual role during macrophage activation. It not only promotes M2 macrophage polarization through the glutamine-UDP-acetylglucosamine pathway and utilizes α-ketoglutarate produced by glutamine catabolism but also mediates glutamine-dependent aging-free and/or γ-aminobutyric acid shunting processes to induce M1 macrophage polarization [88,92,93]. Different polarization processes lead to different effects on macrophage immune function; therefore, glutamine can be pro- or anti-inflammatory in regulating the pathological process of IBD, depending on the different pathological context. The use of alanyl-glutamine therapy was found to inhibit the secretion of Th17-related cytokines by reducing the number of macrophages infiltrating the peritoneal cavity, ultimately ameliorating the inflammatory response [156,157,158]. In contrast, findings have shown that glutamine supplementation upregulates pro-inflammatory cytokines including IL-1β, IL-17, and TNF-α [159]. The above different experimental results may be due to the different doses of glutamine used, hence the data suggesting that low doses of glutamine (0.5% basal diet) significantly promote the expression of pro-inflammatory factors, whereas high doses of glutamine (2% basal diet) inhibit the expression of pro-inflammatory factors [159]. Recent studies have found that the alpha-ketoglutarate/succinate ratio is a key factor in the regulation of macrophage immune metabolism by glutamine. A high value of this ratio promotes M2 macrophage polarization and vice versa for M1 macrophages [93]. Therefore, it is difficult to determine the optimal glutamine dose to improve IBD. In this process, factors such as the pathological course of IBD, the supplemental dose of glutamine, and the metabolic balance between glutamine and other amino acids should be included in the study.

## 6. Therapeutic Approaches Targeting Macrophage Immunometabolism in IBD

Macrophage metabolism is a dynamic process that changes in response to alterations in the tissue microenvironment, and these changes also reflect the different pathologic processes of a given disease. As mentioned earlier, macrophage metabolic processes are significantly altered in IBD, including enhanced glycolysis, impaired FAO processes with altered amino acid metabolic functions.

Tofacitinib is an oral pan-JAK inhibitor that has been approved for the treatment of UC [160,161], and it promotes M2 macrophage polarization in colitis mice by inducing macrophage OXPHOS processes [162,163]. However, some patients have experienced adverse side effects with tofacitinib, such as severe infections (e.g., herpes zoster) and thromboembolism [164,165]. To circumvent the occurrence of these side effects, researchers have developed selective JAK1 inhibitors such as filgotinib [166,167] and ubadatinib [168,169] as alternatives to tofacitinib, and the results of a phase II clinical study have shown that both of these drugs have demonstrated good results in relieving celiac disease and moderate to severe UC [166,167,168,169,170]. However, the mechanism of how these two drugs activate intestinal macrophages and participate in the regulation of their metabolism remains to be further investigated.

Recent studies have also identified other glycolytic regulators other than JAK that can be used to activate macrophages and treat IBD, such as mTORC1, HIF-1α, and AMPK. Mmong these, d-mannose, a naturally occurring C-2 glucose exopolymer, alleviates ulcerative colitis in mice by inhibiting glucose catabolism and succinate-mediated activation of HIF-1α in macrophages [171]. Similarly, it was also shown that spermidine, a natural polyamine, could enhance oxidative phosphorylation and FAO processes through activation of the AMPK/HIF-1α pathway, causing macrophages to exhibit anti-inflammatory properties, ultimately effectively ameliorating colitis in mice [172]. In addition to the endogenous molecules mentioned above, some chemicals present in natural plants can also target the immune metabolism of macrophages to exert ameliorative effects on IBD. Zhuang et al. have clarified that the small molecule adenosine can restore the balance between the M1 and M2 types of macrophages by inhibiting the HIF-1α-dependent glycolysis pathway and, thus, exerts an ameliorative effect on DSS-induced colitis in mice [173]. In addition, Dioscin, a steroidal saponin extracted from the roots of Chrysanthemum pastorale, simultaneously inhibits M1 macrophage polarization by modulating the mTORC1/HIF-1α-dependent glycolytic pathway and promotes M2 macrophage polarization by regulating the mTORC2/PPAR-γ-dependent FAO process, which ultimately ameliorates DSS-induced colitis in mice [174]. Didymin, a dietary citrus flavonoid, ameliorates ulcerative colitis by rebalancing M1/M2 macrophages through 4-hydroxyacyl coenzyme a dehydrogenase β-mediated FAO rather than glycolysis [175].

In addition, reviewing studies of approved drugs with metabolic targets could deepen our understanding of the metabolic adaptations of these drugs and provide new opportunities for better treatment of IBD. It is well known that metformin, a well-established hypoglycemic agent, ameliorates colitis in mice by inhibiting the endogenous fatty acid synthesis/AKT pathway and its downstream MAPK pathway in macrophages [176]. Overall, the above studies suggest that the manipulation of macrophage metabolic pathways and related transcriptional regulators may provide a viable strategy for finding effective targets and potent drugs for the treatment of IBD. It is important to note that many of the metabolic targets found in macrophages may have already been identified in many other cell types. Therefore, combining macrophage-targeted drug delivery technologies, such as nanotechnology, will greatly accelerate the effectiveness of new drug discovery (Table 1).

## 7. Specific Goals and Potential Challenges of Treatment

Research on immune metabolism over the past decade has made significant progress and has clearly demonstrated that the foundation of life is constituted by the interplay of immune metabolism, which is also at the core of numerous diseases such as IBD. In the above description, we highlight the importance of the metabolic fate of nutrients versus microenvironmental stimuli during macrophage polarization. We also summarize potentially effective drugs that target macrophage immune metabolism and thereby treat IBD. As mentioned earlier, recent advances in research on immune metabolism have deepened our understanding of macrophage polarization processes and suggested new ideas for effective therapeutic tools for IBD.

Despite the continuous new advances around these topics, there are still important unanswered questions targeting macrophage immune metabolism in IBD. The first problem is that most of the molecular mechanisms currently used to unravel macrophage immune metabolism have been obtained through in vitro studies, whereas in vivo, the intestinal microenvironment in which macrophages reside is much more complex. For example, various changing cellular components (nutrient concentrations, metabolites, cytokines, transcriptional regulators, etc.) and extracellular factors (oxygen tension, pH, stimuli from other cells or tissues) affect macrophage polarization and their metabolism, but in vitro studies do not mimic this complex tissue microenvironment. The resolution of these issues contributes to a broader analysis of macrophage immunometabolism over tissue/organ or even systemic water. The advanced technologies that are currently available, such as mass spectrometry imaging, multi-omics coanalysis, and single-cell sequencing technologies, will help to piece together a complete picture of macrophage immunometabolism in IBD.

Another issue that exists is that dynamic cellular metabolic processes can respond rapidly to environmental changes under disease. Since the intestinal microenvironment changes differently during the different pathological processes of IBD (e.g., inflammatory or remission phases), the study of macrophage immune metabolism during the multiple phases of IBD would accelerate our understanding of the dynamic metabolic profiles of macrophages and analyze whether these profiles could be effective targets for disease amelioration. Finally, different sources of cells bring different metabolic profiles, and most of the studies carried out on macrophage immune metabolism have been performed with bone marrow or monocyte-derived macrophages. Therefore, in order to better characterize the different metabolic profiles of macrophage immunity in normal and IBD, studies using multiple sources of macrophages, such as monocyte-derived or macrophages from the lamina propria or myofibroblasts, are needed and will be the focus of future research.

## 8. Barriers to Clinical Translation

Although the various types of drugs developed to target macrophages for the treatment of intestinal inflammation have made considerable progress, drug development has been severely constrained by a number of factors such as biological complexity, drug-biological interactions, scale-up effects in the translational process, and the complexity of quality control, which have been the main barriers to low clinical translation rates.

### 8.1. Biological Barriers

Drugs undergo a complex multi-step cascade process in the body to exert their efficacy, including entry into the blood circulation, accumulation to the site of inflammation, penetration into the interior of inflamed tissues, endocytosis, intracellular transport, and drug release. Inefficiency in any step reduces the overall therapeutic effect. However, there are a range of biological barriers present in the body that can prevent the drug from moving efficiently through each of these processes, limiting therapeutic efficacy.

#### 8.1.1. Nano-Biological Interactions in the Bloodstream

The presence of a large number of proteins in the blood may bind to the surface of the drug to form a “protein crown”, thereby altering its physicochemical characteristics and stability and preventing the specific binding of the target molecule to the receptor. In addition, blood protein fractions vary from patient to patient, leading to heterogeneity of the “protein crowns” and unpredictability of their fate in vivo.

#### 8.1.2. Clearance by the Mononuclear Phagocytic System (MPS)

Most drugs are taken up and cleared by macrophages or endothelial cells in the liver or spleen, preventing further delivery to inflammatory tissues. At the same time, non-specific accumulation in healthy tissues can lead to long-term toxicity.

### 8.2. Clinical Safety Issues

Many patients develop severe immune-related adverse reactions after taking the drug, leading to the termination of clinical trials. There is an urgent need to supplement the means of evaluating the safety of drugs with new methods.

### 8.3. Physicochemical Characteristics and Scale-Up Effects

Physicochemical parameters are crucial for the biological effects of drugs, including immune escape, drug penetration, and cellular uptake. The complexity of drug microstructures and compositions, and the multi-step or complex technologies involved in the construction process, result in poor reproducibility of preparation and extreme difficulty in scale-up, which in turn affects in vivo biological effects. Therefore, the production of drugs requires strict control of the physical and chemical properties between batches, with higher requirements for chemistry, manufacturing and control.

### 8.4. Animal Models

The lack of animal models capable of accurately simulating precise human inflammatory bowel disease is also one of the shortcomings, leading to poor correlation between preclinical studies and clinical trial results (pharmacokinetics, biodistribution and safety, etc.) [177].

## 9. Concluding Remarks and Future Perspectives

**Figure 4:**
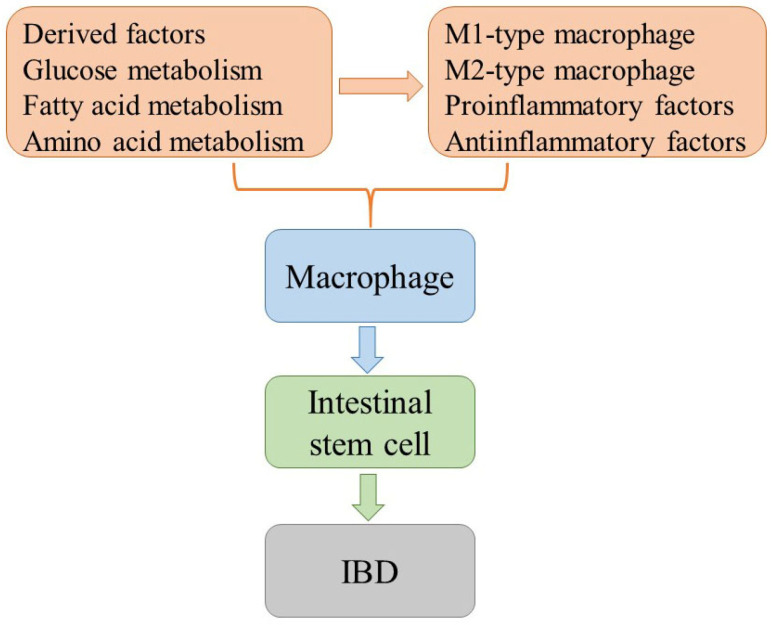
**Illustrates the interaction patterns among IBD, macrophages, and crypt stem cells.** Beyond establishing a vital barrier, these cells also exert control over macrophage function, modulating the immune response by influencing macrophage polarization and participating in defense against pathogens as well as the secretion of cytokines. Macrophages contribute positively to the promotion of intestinal stem cell proliferation through their regulatory role. Stem cells, endowed with self-renewal and differentiation capabilities, play a pivotal role in repairing damaged intestinal epithelium and maintaining homeostasis.

Intestinal homeostasis is a finely orchestrated process in which intestinal epithelial cells, macrophages, and stem cells play vital roles. Intestinal epithelial cells are responsible for maintaining immune tolerance by upholding the integrity of the intestinal barrier and regulating immune responses. Macrophages, on the other hand, are pivotal in the clearance of pathogens and the control of inflammatory reactions. They engage in dynamic interactions with intestinal epithelial cells and other immune cells to maintain a delicate balance within the intestinal immune system while also overseeing the regulation of intestinal stem cells, thus, ensuring the overall stability of intestinal tissue. Stem cells exhibit the remarkable capacity for self-renewal and differentiation along multiple lineages. This continuous generation of intestinal epithelial cells and various other cell types within the intestinal environment is vital for the ongoing repair and regeneration of intestinal tissue. The interactions between intestinal epithelial cells and macrophages serve as a means to regulate the intestinal immune response and modulate the state of inflammation, thereby impacting the integrity of the intestinal barrier function. This intricate web of mutual regulation between macrophages and stem cells, as well as between stem cells and the intestinal epithelium, represents a pivotal step in the repair and regeneration of intestinal tissue (Figure 4).

## Figures and Tables

**Table 1:** Therapeutic approaches targeting macrophage immunometabolism in experimental IBD.

Drug	Target Point	Effect	Machine	References
Tofacitinib	A pan-JAK inhibitor	Increases M2-like macrophages by promoting OXPHOS	Suppressing JAK1 and JAK3	[163]
GLPG0555	A JAK1 selective inhibitor	Increases M2-like macrophages by promoting OXPHOS	Suppressing JAK1	[163]
D-mannose	Monosaccharide	Inhibits pro-inflammatory macrophage IL-1β production by impairing glucose catabolism and succinate-mediated HIF-1α activation	By phosphomannose isomerase	[171]
Spermidine	Polyamine	Promoting M2 polarization by enhancing OXPHOS and FAO fluxes	Blocking AMPK/HIF-1α pathway	[172]
Tiliroside	Flavonoid	Restores M1/M2 balance by blocking glycolysis	Suppressing HIF-1α	[173]
Dioscin	Steroidal saponin	Inhibits M1 by inhibiting aerobic glycolysis; promotes M2 polarization by facilitating FAO	Suppressing mTORC1/HIF-1α pathway and promoting mTORC2/PPAR-γ pathway	[174]
Didymin	Flavonoid	Restoration of M1/M2 balance via promoting FAO	Promoting 4 hydroacyl-CoA dehydrogenase β	[175]
Metformin	A hypoglycemic drug	Inhibition of pro-inflammatory macrophage activation through inhibition of fatty acid synthesis	Suppressing fatty acid synthase/Akt/MAPK pathway	[176]

Abbreviations: OXPHOS, oxidative phosphorylation; JAK, Janus Kinase; HIF-1α, hypoxia-inducible factor-1 alpha; M1: Classical activated macrophages; M2: Alternatively activated macrophages; AMPK, adenosine monophosphate-activated protein kinase; FAO, fatty acid oxidization; mTORC1, mammalian target of rapamycin complex 1; mTORC2, mammalian target of rapamycin complex 2; Akt, protein kinase B; MAPK, mitogen-activated protein kinase; PPAR-γ, peroxisome proliferator-activated receptor gamma.

## Data Availability

Data will be made available on request.

## Abbreviations

IBD: Inflammatory bowel disease; UC: Ulcerative colitis; CD: Crohn’s disease; IEC: Intestinal epithelial cells; MSCs: Mesenchymal stem cells; HSCs: Hematopoietic stem cells; ISC: Intestinal stem cells; BMT: Bone marrow transplantation; MHC: Histocompatibility complex class; GVHD: Graft-versus-host disease; TNF: Tumor necrosis factor; TH: T helper; Treg: Regulatory T; hIOs: Human pluripotent stem cell-derived intestinal organoids; ILCs: Innate lymphoid cells; ER: Endoplasmic reticulum; DDR: DNA damage response; CSCs: Colonic stem cells; ILC2: Group 2 innate lymphoid-like cells; TCA: Tricarboxylic acid cycle; PPP: Pentose-phosphate pathway; FAO: Fatty acid oxidation; OXPHOS: Oxidative phosphorylation; PGE2: Prostaglandin E2; LGG: *Lactobacillus rhamnosus* GG; CSF1: Colony-stimulating factor; GLUT1: Glucose transporter 1; PFKFB3: 6-phosphofructo-2-kinase/fructose-2,6-bisphosphatase; DSS: Dextran sulfate sodium salt; SUCNR1: Succinate receptor 1; PPAR-γ: Peroxisome proliferator-activated receptor gamma.
